# Enhanced Electrical Conductivity and Seebeck Coefficient in PEDOT:PSS via a Two-Step Ionic liquid and NaBH_4_ Treatment for Organic Thermoelectrics

**DOI:** 10.3390/polym12030559

**Published:** 2020-03-03

**Authors:** Jonathan Atoyo, Matthew R. Burton, James McGettrick, Matthew J. Carnie

**Affiliations:** SPECIFIC-IKC, Materials Research Centre, College of Engineering, Swansea University, Bay Campus, Swansea SA1 8EN, UK; 922825@swansea.ac.uk (J.A.); m.r.burton@swansea.ac.uk (M.R.B.); j.d.mcgettrick@swansea.ac.uk (J.M.)

**Keywords:** conducting polymers, PEDOT:PSS thermoelectric, organic thermoelectric, enhanced power factor

## Abstract

A two-step approach of improving the thermoelectric properties of Poly(3,4-ethylenedioxythiophene)poly(4-styrenesulfonate) (PEDOT:PSS) via the addition of the ionic liquid, 1-Ethyl-3-methylimidazolium bis(trifluoromethylsulfonyl)imide (EMIM:TFSI) and subsequent reduction with NaBH_4_ is presented. The addition of 2.5 v/v% of EMIM:TFSI to PEDOT:PSS increases the electrical conductivity from 3 S·cm^−1^ to 1439 S·cm^−1^ at 40 °C. An additional post treatment using the reducing agent, NaBH_4_, increases the Seebeck coefficient of the film from 11 µV·K^−1^ to 30 µV·K^−1^ at 40 °C. The combined treatment gives an overall improvement in power factor increase from 0.04 µW·m^−1^·K^−2^ to 33 µW·m^−1^·K^−2^ below 140 °C. Raman and XPS measurements show that the increase in PEDOT:PSS conductivity is due to PSS separation from PEDOT and a conformational change of the PEDOT chains from the benzoid to quinoid molecular orientation. The improved Seebeck coefficient is due to a reduction of charge carriers which is evidenced from the UV–VIS depicting the emergence of polarons.

## 1. Introduction

Organic thermoelectric generators (OTEGs) are solid state devices that can convert heat energy into electrical energy [1,2]. OTEGs can therefore be useful in wearable devices due to their properties such as their light weight, flexibility and solution processability [3,4]. However, to be commercially viable these materials are required to exhibit high thermoelectric performance which is usually described by the dimensionless figure of merit, ZT equation [2,5].

ZT = S^2^σ T/κ (1)

where S is the Seebeck coefficient, σ is the electrical conductivity, T is the absolute temperature and κ is the thermal conductivity [6]. Poly(3,4-ethylenedioxythiophene)poly(4-styrenesulfonate) (PEDOT:PSS) has been widely studied as an OTEG material, which is in part due to its low thermal conductivity 0.1–0.3 W·m^−1^·K^−1^ [7,8] and tuneable doping states from chemical treatment [9]. Properties such as, water solubility, processability, ease of printing and device fabrication, as well as miscibility with polar solvents have made PEDOT:PSS a common starter material in OTEG research [10,11]. However, due to the pristine PEDOT:PSS exhibiting low electrical conductivity, σ (≈ 2 S·cm^−1^) and Seebeck coefficient, S (≤ 14 μV·K^−1^), the resultant Power Factor (PF = S^2^ × σ) is too low to make pristine PEDOT:PSS a commercially viable thermoelectric material [12]. In order to improve the thermoelectric performance of PEDOT:PSS, effective chemical treatment is required to optimize the PF. It is possible to significantly increase the electrical conductivity of PEDOT:PSS via treatment with polar solvents, acids and salt solutions [13,14]. Conductivities of 620, 640, 800, 1300 and 1900 S·cm^−1^ have been achieved by treating PEDOT:PSS with dimethyl sulfoxide (DMSO), ethylene glycol, polyethylene glycol, methanol and formic acid, respectively [15,16]. Solvent treatments, however, do not significantly affect the Seebeck coefficient of PEDOT:PSS. Nonetheless, due to the increased electrical conductivity, the derived PF from solvent treatment is significantly higher than pristine PEDOT:PSS. The solvent treatment mechanism also has an influence on thermoelectric performance, for example, DMSO treatment of the PEDOT:PSS suspension yields a PF of 18.2 μW·m^−1^·K^−2^ while post treatment of the PEDOT:PSS film gives a PF of 30.1 μW·m^−1^·K^−2^ [17]. 

In 2007, the first study to determine the effects of ionic liquid (IL) treatment on the electrical conductivity of PEDOT:PSS was conducted by utilizing five different ionic liquids [18]. The study used, 1-butyl-3-methylimidazolium bromide (BMIM:Br), 1-benzyl-3-methylimidazolium chloride (BzMIM:Cl), 1-butyl3-methylimidazolium tetrafluoroborate (BMIM:BF4) and 1-butyl-1-methylpyrrolidium chloride (BMPro:Cl) and 1 ethyl-3-methylimidazolium chloride (EMIM:CL). The BMIM:BF_4_ treated film achieved the highest conductivity of 136 S·cm^−1^. More recently, [19] significantly higher electrical conductivity has been shown by controlling molecular ordering in PEDOT:PSS chains by using 1-ethyl-3-methylimidazolium tetracyanoborate (EMIM:TCB), achieving an electrical conductivity of 2103 S·cm^−1^. PEDOT:PSS–ionic liquid composites have also exhibited resistance to mechanical deformation while maintaining high performance [8]. These properties of PEDOT:PSS–ionic liquid composites make them particularly suitable candidates for printing onto flexible substrates and textiles because they can withstand shape deformation that could occur in wearable electronics. 

Whilst solvent and ionic liquid treatments leave Seebeck coefficient largely unchanged, treatment with base solutions and reducing agents can tune the oxidation state of PEDOT and thus the Seebeck coefficient can be improved [20]. NaOH solution treatment had been used to increase the Seebeck coefficient from 12.6 μV·K^−1^ to 54 μV·K^−1^ in pristine PEDOT:PSS, however, the treatment lead to a decrease in electrical conductivity from 837 S·cm^−1^ to 0.04 S·cm^−1^. Only dual solvent/base treatment by mixing a DMSO and NaOH produced a significant increase in PF of 33 μW·m^−1^·K^−2^ [21]. Seebeck coefficients of 104 μV·K^−1^ have been achieved in NaBH_4_ treated PEDOT:PSS, but resulted in a reduction of the electrical conductivity from 716 to 11 S·cm^−1^ [21]. Hydrazine has also been utilized and a PF of 112 μW·m^−1^·K^−2^ has been achieved by treating the PEDOT:PSS with a hydrazine in DMSO solution yielding an electrical conductivity of 578 S·cm^−1^ and Seebeck coefficient was 67 μV·K^−1^ [14]. 

To the best of our knowledge there have been no investigations looking at the effects of a reducing agent treatment on a thin film PEDOT:PSS–ionic liquid composite. In this study we report on a 1-Ethyl-3-methylimidazolium bis(trifluoromethyl sulfonyl)imide (EMIM:TFSI): PEDOT:PSS composite and the subsequent post treatment with NaBH_4_-DMSO solution. The PEDOT:PSS ionic liquid composite has superior electrical conductivity and unaffected Seebeck coefficient. The reducing agent NaBH_4_ is utilized as a post treatment to tune the oxidation state and improve the Seebeck coefficient. This study also explores the mechanisms responsible for the improved electrical conductivity and Seebeck coefficient.

## 2. Materials and Methods 

### 2.1. Materials Used

All chemical reagents used were purchased from (Sigma-Aldrich, Gillingham, England, United Kingdom). The chemicals used in this study are NaBH_4_ powder 99% Reagent grade, EMIM:TFSI ionic liquid 98% HPLC grade, DMSO anhydrous liquid 99.9%. The PEDOT:PSS was Heraeus Clevios PH1000.

### 2.2. Fabrication of PEDOT:PSS EMIM:TFSI Composite Films

A 1 mm thick plain nonconductive glass was cut into 2.2 cm by 2.2 cm pieces and washed in a Helmanax solution. The glass substrates were then rinsed with deionised water and subsequently rinsed with acetone and then isopropanol. The glass was then dried in a stream of nitrogen gas. The PEDOT:PSS dispersion (3000 µL) was placed into 7 mL glass vials. Aliquots of 15, 30, 45, 60 and 75 µL of 1-Ethyl-3-methylimidazoliumbis(trifluoromethyl sulfonyl)imide (EMIM:TFSI) were added to separate vials to give 0.5%, 1%, 1.5%, 2% and 2.5% (v/v) dispersions. The vials were shaken using a vortex mixer then heated at 120 °C for 3 min. Then 300 µL of the PEDOT:PSS/EMIM:TFSI solutions were then pipetted onto a pre-cleaned glass substrate and spin-coated at 2000 rpm with an acceleration speed of 2000 rpm·s^−1^ for 30 s, then annealed at 120 °C in air for 10 min. The films produced were 60–70 nm, as determined by profilometry.

### 2.3. Fabrication of PEDOT:PSS EMIM:TFSI-NaBH_4_ Composite Films

To make the 1% (w/v) NaBH_4_-DMSO reducing agent solution, 20 mg of NaBH_4_ was dissolved in 2 mL of DMSO by shaking it using a vortex mixer and heating at 120 °C for 5 min. Then 200 µL of the NaBH_4_ solution was drop cast on to the PEDOT:PSS-EMIM:TFSI films at room temperature for 1 min and then rinsed with acetone. The films were the dried in dry nitrogen gas before annealing at 120 °C for 5 min. The films thickness remained relatively unchanged after NaBH_4_ treatment (55–65 nm).

### 2.4. Characterization

Film thicknesses were determined using a Dektak 150 stylus profilometer(Veeco Instruments Inc, New York, USA), with all films measuring between 55 nm and 70 nm. Silver conductive paint was used to create top and bottom contacts for the Seebeck and electrical measurements which were conducted on a ULVAC ZEM-3 system (ADCANCE RIKO, Yokohama, Japan), under a helium atmosphere (see Figure S4 for measurement set up). XPS was carried out using a Kratos Axis Supra (Kratos Analytical), (Kratos Analytical Ltd, Manchester, United Kingdom), using a monochromated Al Kα source. The UV–VIS absorbance spectra were measured with a Perkin Elmer Lambda 750 spectrophotometer (PerkinElmer, Shelton, USA), using an uncoated 1 mm thick glass substrate as a reference sample. The spectrum was run from 400 nm to 2400 nm. The Raman spectra were measured using a Renishaw inVia Raman system (Renishaw, Gloucestershire, United Kingdom). A 532 nm laser and a 50× objective lens were used to measure the Raman spectra of the samples with an acquisition time of 10 s.

## 3. Results and Discussion

### 3.1. Thermoelectric Performance of PEDOT:PSS EMIM:TFSI Composite Films

**Figure 1:**
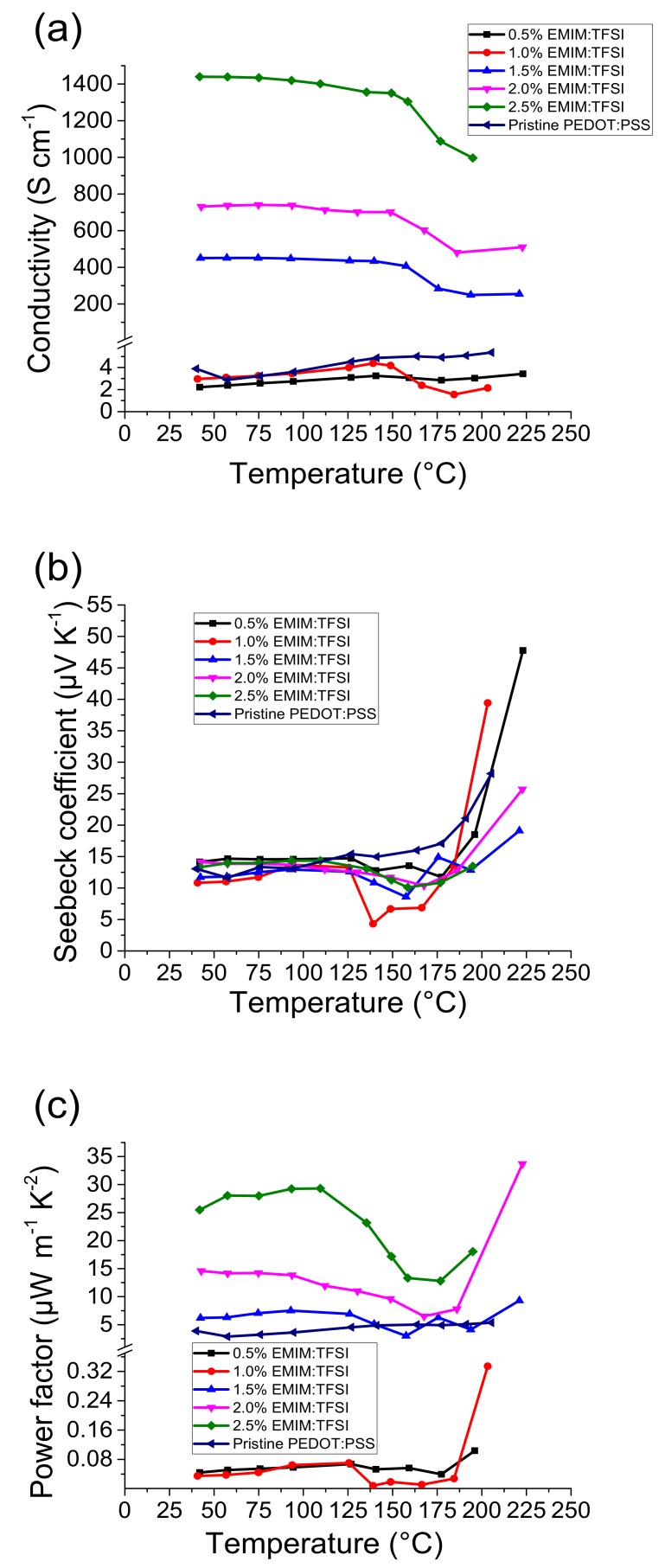
Thermoelectric properties of 1-Ethyl-3-methylimidazolium bis(trifluoromethylsulfonyl)imide (EMIM:TFSI) treated films, **a**) the electrical conductivity, **b**) Seebeck coefficient and **c**) power factor.

Figure 1a shows the electrical conductivity of PEDOT:PSS and EMIM:TFSI treated PEDOT:PSS films according to the solution v/v percentage from which they were produced. 

When EMIM:TFSI treated films were manufactured from v/v% solutions greater than 1.5%, there is a significant increase in electrical conductivity. 

This concentration based increment in electrical conductivity is also observed in other PEDOT:PSS ionic liquid composites [22]. The electrical conductivity of pristine PEDOT:PSS at 40 °C was measured as 3.4 S·cm^−1^ which is in accordance to literature values [10,20]. When EMIM:TFSI was added into PEDOT:PSS solutions at 0.5% and 1%, there were no significant changes to the electrical conductivities of the films. 

The 1.5% EMIM:TFSI film, however, exhibited an increase in electrical conductivity to 451 S·cm^−1^. This is higher than the values obtained at the same concentration using other ionic liquids such as BMIM:TFB (136 S·cm^−1^) and BMIM:CL (49 S·cm^−1^) [18]. EMIM:TCB however, yields a slightly higher electrical conductivity at the same concentration (500 S·cm^−1^) [22].

When increasing the concentration of EMIM:TFSI to 2.0% the electrical conductivity at 40 °C increased further to 737 S·cm^−1^ and further still to 1439 S·cm^−1^ at 2.5%, which is highly conductive when compared to solvent treated films such as DMSO (100–600 S·cm^−1^), [5,23,24] and ethylene glycol (400–1000 S·cm^−1^) [5,16]. EMIM:TCB, in comparison, can yield conductivities of up to 2104 S·cm^−1^ [5,22].

Whilst EMIM:TFSI treated films in comparison yield a slightly lower electrical conductivity at 40 °C, when the temperature is elevated the electrical conductivity for ≥1.5% EMIM:TFSI films exhibit a lower drop in electrical conductivity. Although the electrical conductivity improves significantly with the addition of EMIM:TFSI, it was not possible to increase the concentration beyond 2.5% due to the formation of coagulates which impleaded spin coating.

Figure 1b shows that EMIM:TFSI treatment did not significantly change the Seebeck coefficient of the composite films. Figure 1c shows that the resultant power factors for the 1.5, 2.0 and 2.5% EMIM:TFSI films are significantly higher than PEDOT:PSS across the temperature range studied. The PF for the Pristine PEDOT:PSS at 40 °C is 0.04 µW·m^−1^·K^−2^ and peaks at 0.1 µW·m^−1^·K^−2^ at 177 °C. 2.5% EMIM:TFSI at 40 °C, however, has a PF of 28 µW·m^−1^·K^−2^. This is comparable to optimized post DMSO and ethylene glycol treatments [5]. The PF reaches an optimum at 109 °C of 29 µW·m^−1^·K^−2^. The 1.5 and 2.0% films show a PF of 6 µW·m^−1^·K^−2^ and 14 µW·m^−1^·K^−2^, respectively at 40 °C, whilst the 0.5 and 1% EMIM:TFSI films exhibit a similar PF to Pristine PEDOT:PSS due to their equally low electrical conductivity with negligible increases in Seebeck coefficients.

### 3.2. Post Treatment of EMIM:TFSI Films with NaBH_4_

**Figure 2:**
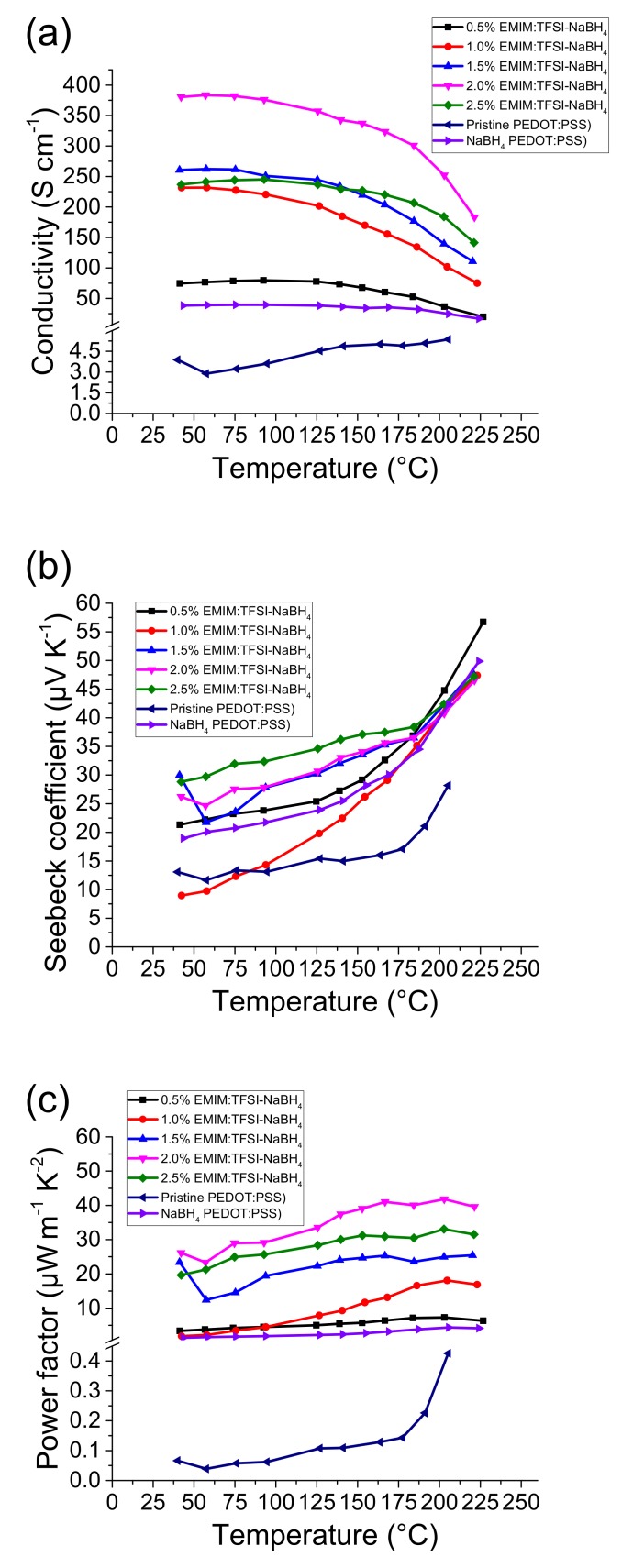
Thermoelectric data for EMIM:TFIS films treated with the 1% w/v NaBH_4_ solution, (**a**) denotes the electrical conductivity, (**b**) depicts the Seebeck coefficient and (**c**) the power factor.

Five PEDOT:PSS/EMIM:TFSI composite films were post treated with a 1% w/v solution of NaBH_4_ in DMSO for 1 min at room temperature plus a control treatment of pure DMSO found at Figure S1. Figure 2a shows that NaBH_4_ treatment reduces the expected electrical conductivity of the EMIM:TFSI composite films. The highest electrical conductivity observed for the EMIM:TFSI-NaBH_4_ films was 380 S·cm^−1^ at 40 °C for the 2.0% films. Although it is expected for the 2.5% film to have higher electrical conductivity, this can be explained due to the treatment mechanism. 

When treated with the reducing agent the kinetics may vary slightly due to difficulty control redox of thiophene molecules, hence why the small difference in electrical conductivity between the 2.0% and 2.5% film.

Figure 2b depicts the Seebeck coefficient data for the NaBH_4_ treated films. Pristine PEDOT:PSS treated with NaBH_4_ has a Seebeck coefficient of 20 µV·K^−1^, which is similar to values seen in the literature [25]. This is double that of untreated EMIM: PEDOT:PSS composite films, as shown here and by others [26,27,28]. It is of note that the electrical conductivity for the NaBH_4_ treated PEDOT:PSS film (Figure 2a) is higher than PEDOT:PSS. This is due to DMSO in the NaBH_4_ solution.

The DMSO, and EMIM:TFSI-DMSO, are present in Supplementary InformationSupplementary Information Figure S1, in which it is shown that the presence of DMSO does not affect the Seebeck coefficient.

The 1% EMIM:TFSI, NaBH_4_ treated film (Figure 2b) exhibits the least significant increase in Seebeck coefficient across the temperature range, as it starts at 9.7 µV·K^−1^ at 40 °C and reaches a Seebeck coefficient of 42 µV·K^−1^ at 200 °C. This is significantly higher, however, than the non-NaBH_4_ treatment sample (Figure 1b). A more pronounced increase in the Seebeck coefficient across the temperature range is observed for the 0.5% EMIM:TFSI NaBH_4_ film, which begins at 22 µV·K^−1^ at 40 °C and steadily rises to 56 µV·K^−1^ 220 °C. A yet more pronounced increase in the Seebeck coefficient is seen in the 1.5, 2.0 and 2.5% EMIM:TFSI NaBH_4_ composite films. The highest Seebeck coefficient observed from the films across the temperature range is attributed to the 2.5% EMIM:TFSI NaBH_4_. At 40 °C the film exhibits a Seebeck coefficient of 30 µV·K^−1^ which increases to a maximum of 33 µV·K^−1^ above 200 °C. It is of note that the presence of the EMIM:TFSI in NaBH_4_ treated PEDOT:PSS films, causes a larger increase in the Seebeck coefficient compared to PEDOT:PSS films treated with NaBH_4_ alone. 

The optimal PF (Figure 2c) is seen for 2% EMIM:TFSI-NaBH_4_ which is 26 µW·m^−1^·K^−2^ at 40 °C and reaches a maximum of 42 µW·m^−1^·K^−2^ at 200 °C. The PF is comparable to the 2.5% EMIM:TFSI (Figure 1c) at lower temperatures, however above 120 °C the PF is significantly higher. This shows the potential to further increase thermoelectric performance of EMIM:TFSI ionic liquid films with reduction via NaBH_4._

### 3.3. Mechanisms for the Improved Electrical Conductivity of EMIM:TFSI and EMIM:TFSI NaBH_4_ Films

**Figure 3:**
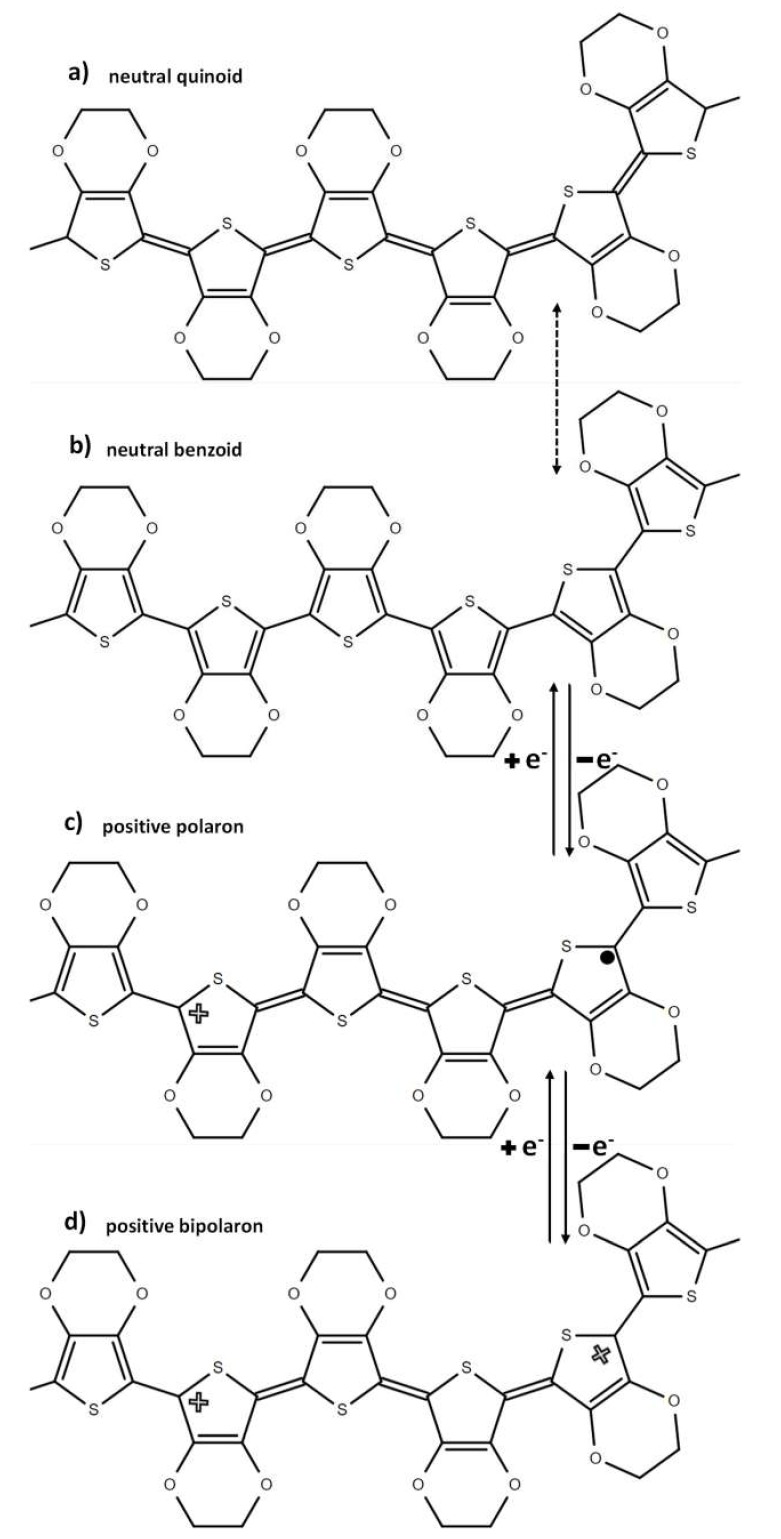
The resonance forms of Poly(3,4-ethylenedioxythiophene) (PEDOT) thiophene back bone, (**a**) denotes the quinoid form, meanwhile (**b**) is the benzoid form. (**c**) and (**d**) represent the positive polaron and bipolaron phase, respectively.

**Figure 4:**
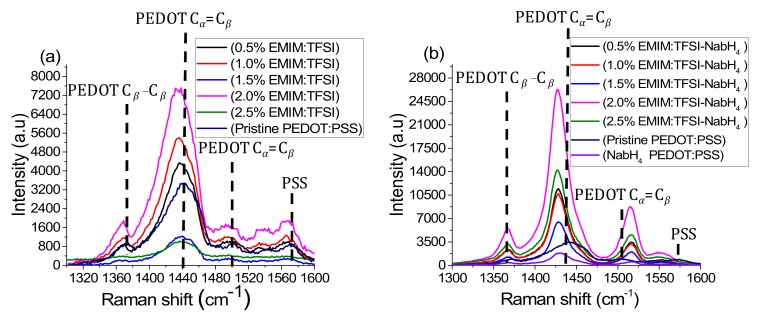
Raman spectroscopy of all films in this study, (**a**) denotes the EMI:TFSI treated films from 0% to 2.5% concentrations, meanwhile (**b**) corresponds to the NaBH_4_-DMSO solution treatment on the 0% to 2.5% EMIM:TFSI films.

The improved electrical conductivity in the EMIM:TFSI and EMIM:TFSI-NaBH_4_ films relative to pristine PEDOT:PSS can be explained by 2 mechanisms. The first mechanism is well established for polar solvents and arises from the conformation change in the PEDOT:PSS back bone from a coiled benzoid structure to a linear/expanded coil quinoid structure, as presented in Figure 3. The interaction among linear PEDOT chains will understandably be stronger than coiled chains [29]. Raman spectroscopy can be utilized to indicate which structure (benzoid or quinoid) is dominant in the thiophene backbone via the position of the symmetrical stretching of the C_α_ = C_β_ typically found at 1440 cm^−1^ [30,31]. A red shift is linked to a benzoid to quinoid shift which can be observed in all EMIM:TFSI composite films as observed in Figure 4a, as well as the EMIM:TFSI-NaBH_4_ composite films as expressed in Figure 4b. The EMIM:TFSI-NaBH_4_ films presented in Figure 4b show the most significant red shift, most likely due to NaBH_4_ treatment. This is because Figure S3 shows 2.0% and 2.5% EMIM:TFSI-DMSO, films with a less significant red shift than films with NaBH_4_ treated films. The quinoid structure as shown in Figure 3a has restricted rotation around the sp^2^ carbon double bond on the thiophene backbone thereby adopting a planer structure with more crystalline packing relative to the benzoid (Figure 3b) structure in which more thiophene units adopt a helical structure with lower molecular ordering that impedes hopping of charge carriers (polarons) within and between the polymer chains [32,33].

Despite the significant red shift (indicating improved carrier mobility) in NaBH_4_ films, the conductivity for these films are lower than EMIM:TFSI films due to reduced charge carriers. Pristine PEDOT:PSS is included in Figure 4 as a baseline. The peaks at 1365 cm^−1^ and 1500 cm^−1^ correspond to the C_β_-C_β_ inter ring stretching vibration and the asymmetrical C_α_=C_β_ vibrations, respectively [30,31]. PSS bond stretching and bending vibrations can be seen at 1580 cm^−1^.

**Figure 5:**
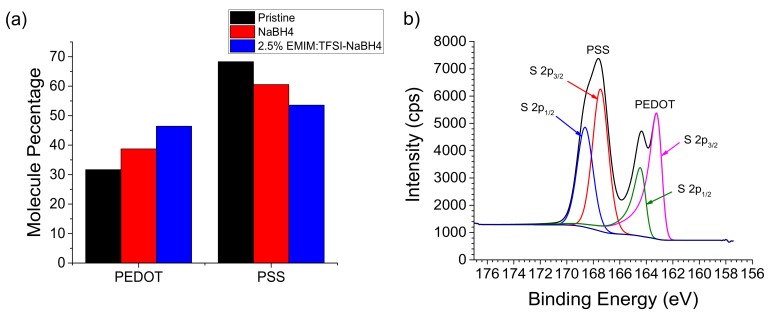
Quantified molecular percentage ratio between PEDOT and poly(4-styrenesulfonate) (PSS) (**a**), and 2.5% EMIM:TFSI-NaBH_4_ XPS spectra outlining deconvoluted sulphur peaks representing PEDOT and PSS (**b**).

The increase in electrical conductivity is also in part due to a second mechanism attributed to a selective removal of PSS over PEDOT. Internal treatment with ionic liquids on PEDOT:PSS where PSS is selectively removed over PEDOT leads to PEDOT chain reorientation to a more conductive linear form [18,22,34,35]. This selective PSS removal is evidenced by XPS quantification shown in Figure 5a. Detailed quantification can be seen in Table S1. 

The concentration of PSS is roughly double that of PEDOT in pristine PEDOT:PSS, which is similar to published results [36,37]. After EMIM:TFSI-NaBH_4_ treatment the PEDOT and PSS quantities are seen to be near equal.

Figure 5b depicts a 2.5% EMIM:TFSI-NaBH_4_ treated film with peak fitting consistent with literature values [38]. Pristine PEDOT:PSS, NaBH_4_ and 2.5% EMIM:TFSI films can be seen in Figure S2. Due to the sulphite sulphur in PSS being bonded to highly electronegative oxygen atoms, its peak is at a higher binding energy than the thiophene sulphur in PEDOT which is bonded to the less electronegative sp^2^ hybridized carbons [39,40]. TFSI is bonded to carbon with electronegative fluorides attached which leads to the sulfonyl sulphur being at a higher still binding energy, as seen in Figure S2. 

As shown in Table S1 and Figure S2, the 2.5% EMIM:TFSI film shows no PEDOT or PSS peak. The peaks at binding energies of 169.02 and 170.23 eV correspond to the TFSI sulphur spin-orbital coupling (S 2p_3/2_) and (S 2p_1/2_) respectively [41]. XPS has a penetration depth of approximately 10 nm, which shows at least the surface of the 60 nm EMIM:TFSI composite films were covered with the TFSI anion. Therefore, to determine the effects of EMIM:TFSI treatment on the PEDOT to PSS ratio, the quantified data for the 2.5% EMIM:TFSI-NaBH_4_ can be used as a proxy for the 2.5% EMIM:TFSI film because they have the same chemical composition with the exception of that the TFSI anion has been washed away from the surface by the NaBH_4_ treatment thereby leading to PEDOT and PSS peaks being observed.

In order to determine that the effects on the PEDOT to PSS ratio are not from DMSO which is present in the NaBH_4_ solution, a NaBH_4_ treated pristine film is shown in Figure 5a. This is because DMSO is known to induce selective removal of PSS in literature [17,42]. EMIM:TFSI-NaBH_4_ films are shown to have a higher proportion of PEDOT than solely NaBH_4_ treated films, therefore it can be concluded that EMIM:TFSI selectively removes PSS over PEDOT. 

### 3.4. Mechanisms for the Improved Seebeck Coefficient of EMIM:TFSI NaBH_4_ Films

An increase in the Seebeck coefficient of PEDOT:PSS can often be attributed to a reduction of carrier concentration [11,13,14]. Treatment with NaBH_4_ results in a redox reaction with PEDOT:PSS as expressed in the equation:[21]

2NaBH_4_ + PEDOT^2+^+2PSS^−^ → 2NaPSS + PEDOT+2BH_3_ + H_2_ (2)

As evidenced by Figure 4, the reduced carrier concentration via NaBH_4_ treatment (Figure 4b) can be observed by a reduction of the width and increase in the intensity corresponding to the symmetric and asymmetric C_α_=C_β_ stretching of the Raman spectra for relative to the films not treated with NaBH_4_ (Figure 4a). This however does not increase the intensity of the PSS region thereby showing no reduction taking place at this region [25].

**Figure 6:**
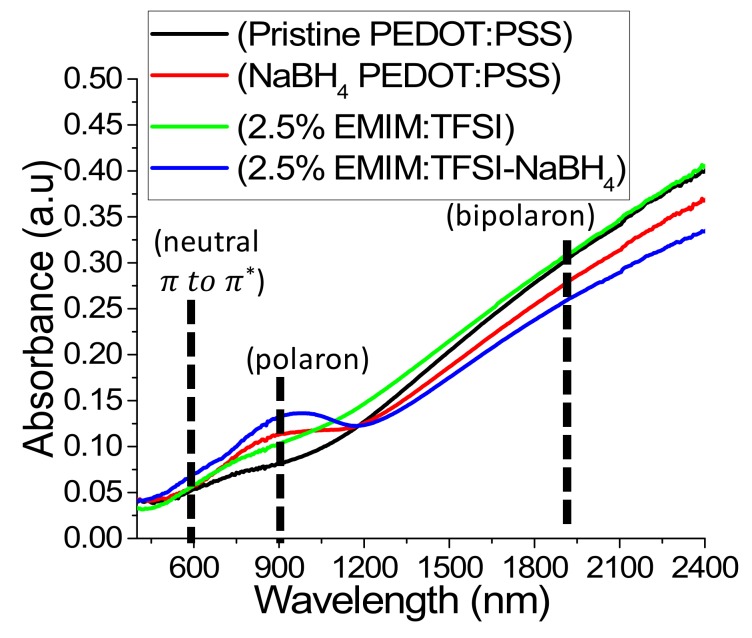
UV–VIS depicting absorbance Pristine PEDOT:PSS, NaBH4 PEDOT:PSS, 2.5% EMIM:TFSI and 2.5% EMIM:TFSI-NaBH_4_ films.

The redox reaction can result in the change of the resonance form of the PEDOT which can be evidenced by a change in the UV-Vis-NIR absorption spectrum. Figure. 6 shows the UV-Vis spectra of a NaBH_4_ treated film in relation to virgin PEDOT:PSS. The NaBH_4_ film shows an emergence of a polaron peak at 900 nm (see also Figure 3c) and a reduction in the peak extending into the NIR compared to pristine PEDOT:PSS, indicating a reduction in the quantity of bipolarons (see also Figure 3d) and an increase of the quantity of polarons in the sample [13,14,21,43]. This explains the increase of Seebeck coefficient seen in Figure 2b for NaBH_4_ treated PEDOT:PSS films. A study utilizing EMIM:DCA discovered that DCA had a reducing effect thereby increasing the Seebeck coefficient by reducing the charge carrier concentration [8]. Figure 1b reveals, however, that the Seebeck coefficient is not significantly affected by EMIM:TFSI treatment. This is consistent with the UV-VIS spectra as depicted in Figure 6, which shows that the 2.5% EMIM:TFSI film has no emergence of a polaron peak at 900 nm or a reduction of the bipolaron region in the NIR. 2.5% EMIM:TFSI-NaBH_4_ films, however, show a more distinct peak in the polaron region and a more reduced bipolaron section compared to the NaBH_4_ treated pristine PEDOT:PSS films, indicating a stronger reduction in those films. This observation is supported by the larger Seebeck coefficients observed in NaBH_4_ treated EMIM:TFSI films, as seen in Figure 2b. 

To understand why the EMIM:TFSI-NaBH_4_ films have higher Seebeck coefficient than the NaBH_4_ PEDOT:PSS, it’s important to note that whilst the ionic liquid has been confirmed to not affect the Seebeck coefficient, the ionic liquid treatment causes phase separation and the subsequent removal of PSS from the polymer. Under the same conditions when post treated with NaBH_4_-DMSO solution, therefore, the reducing agent is in the presence of a higher concentration of PEDOT chains thereby making reduction more effective, due to lower PSS concentration. This consequently leads to a higher Seebeck coefficient.

**Figure 7:**
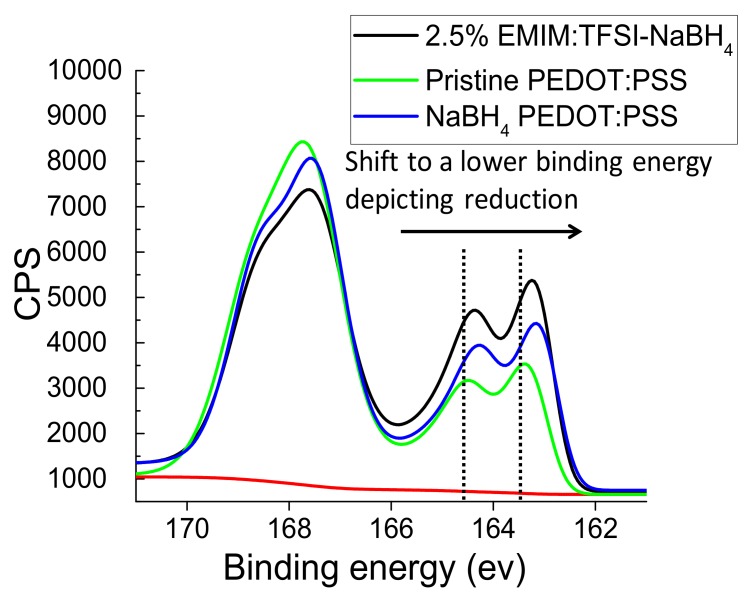
Overlaid XPS spectra showing a lower binding energy shift for NaBH_4_ treated films relative to Pristine PEDOT:PSS indicating reduction.

Figure 7 depicts the overlaid XPS spectra for Pristine PEDOT:PSS, NaBH_4_ PEDOT:PSS and 2.5% EMIM:TFSI NaBH_4_. The XPS data shows a chemical shift in the PEDOT region for the NaBH_4_ treated films to a lower binding energy showing reduction of some thiophene units from bipolarons to polarons has occurred, which agrees with the UV–VIS observations. Figure 7 in conjunction with Table S1 and Figure 5b show the PEDOT spin-orbital sulphur coupling for the Pristine PEDOT:PSS (S 2p_3/2_) shifts from a binding energy of 163.2 eV to 163.0 eV when treated with NaBH_4_. The 2.5% EMIM:TFSI-NaBH_4_ film shows the same shift from 163.2 eV for Pristine PEDOT:PSS (S 2p_3/2_) to 163.0 eV. A similar shift can be observed for the (S 2p_1/2_) peak. This shift is also observed in literature for reducing agent treatment on PEDOT:PSS [14,25].

## 4. Conclusions

A thin film PEDOT: PSS ionic liquid composite utilizing the ionic liquid EMIM:TFSI was synthesized with superior thermoelectric properties to Pristine PEDOT:PSS. The ionic liquid significantly improved the electrical conductivity from 3 S·cm^−1^ to 1439 S·cm^−1^ for the 2.5% EMIM:TFSI film thereby leading to a PF of 28 µW·m^−1^·K^−2^ at 40 °C. A further post treatment with NaBH_4_-DMSO solution was used to improve the Seebeck coefficient which lead to an increase in the PF for most of the films treated with this solution relative to the ones treated with only the ionic liquid. At above 140 °C the 2.0% EMIM:TFSI NaBH_4_ film was optimized to a PF of 40 µW·m^−1^·K^−2^, meanwhile the 2.5% EMIM:TFSI NaBH_4_ was optimized to 33 µW·m^−1^·K^−2^ at the same temperature. 

This simple two-step process is fully solution processable and shows the potential for further optimization of the PF of PEDOT:PSS ionic liquid composites by fine tuning the concentration of the NaBH_4_-DMSO solution and finding more precise ways to control the redox of PEDOT films to minimize the depletion of electrical conductivity. Raman showed that there was a conformational change from benzoid to quinoid. XPS confirmed a second mechanism for improved electrical conductivity via a phase separation and the selective removal of PSS. The improved Seebeck coefficient was confirmed to be due to the post treatment with NaBH_4_-DMSO solution, however, it was discovered that the ionic liquid treated films had higher Seebeck coefficient. The study proved that this discrepancy was due to the ionic liquid removing PSS and exposing the NaBH_4_-DMSO solution to more PEDOT chains, hence causing further reduction. The UV–VIS and XPS provided evidence for a reduction in charge carriers from bipolarons to polarons with the NaBH_4_-DMSO solution treatment. Changing the synthesis method whereby the coagulation of the solution of PEDOT:PSS ionic liquid does not occur until a higher ionic liquid concentration may prove helpful in optimizing the electrical conductivity of these films, as the results showed a positive correlation between concentration of ionic liquid and electrical conductivity.

## Supplementary Materials

The following are available online at https://www.mdpi.com/2073-4360/12/3/559/s1, Figure S1: Depicting thermoelectric data of EMIM:TFSI, EMIM:TFSI-NABH4 and DMSO controls; Figure S2: The deconvoluted XPS spectra of 4 films, (a) denotes 2.5% EMIM:TFSI film, (b) denotes Pristine PEDOT:PSS, (c) corresponds to the NaBH4 Pristine PEDOT:PSS film, meanwhile (d) corresponds to the 2.5% EMIM:TFSI NaBH4 film; Figure S3: Raman spectra depicting ionic liquid films treated with DMSO, post treatment without NaBH4 to show that NaBH4 presence is the major cause for the increased red shift; Figure S4: ZEM-3 set up depicting electrodes and film measurement for Seebeck coefficient and electrical conductivity; Table S1: Sulphur 2p peak position and quantification data.

## References

[1] Bubnova O., Crispin X. (2012). Towards polymer-based organic thermoelectric generators. Energy Environ. Sci..

[2] Cowen L.M., Atoyo J., Carnie M.J., Baran D., Schroeder B.C. (2017). Review—Organic Materials for Thermoelectric Energy Generation. ECS J. Solid State Sci. Technol..

[3] Wang H., Yu C. (2019). Organic Thermoelectrics: Materials Preparation, Performance Optimization, and Device Integration. Joule.

[4] Culebras M., Choi K., Cho C. (2018). Recent Progress in Flexible Organic Thermoelectrics. Micromachines.

[5] Wei Q., Mukaida M., Kirihara K., Naitoh Y., Ishida T. (2015). Recent progress on PEDOT-based thermoelectric materials. Materials.

[6] Zhang B., Sun J., Katz H.E., Fang F., Opila R.L. (2010). Promising thermoelectric properties of commercial PEDOT:PSS materials and their Bi2 Te3 powder composites. ACS Appl. Mater. Interfaces.

[7] Smith P.M., Su L., Gong W., Nakamura N., Reeja-Jayan B., Shen S. (2018). Thermal conductivity of poly(3,4-ethylenedioxythiophene) films engineered by oxidative chemical vapor deposition (oCVD). RSC Adv..

[8] Kee S., Kim H., Paleti S.H.K., El Labban A., Neophytou M., Emwas A.-H., Alshareef H.N., Baran D. (2019). Highly Stretchable and Air-Stable PEDOT:PSS/Ionic Liquid Composites for Efficient Organic Thermoelectrics. Chem. Mater..

[9] Huang J., Miller P.F., Wilson J.S., de Mello A.J., de Mello J.C., Bradley D.D.C. (2005). Investigation of the effects of doping and post-deposition treatments on the conductivity, morphology, and work function of poly(3,4-ethylenedioxythiophene)/poly(styrene sulfonate) films. Adv. Funct. Mater..

[10] Sun K., Zhang S., Li P., Xia Y., Zhang X., Du D., Isikgor F.H., Ouyang J. (2015). Review on application of PEDOTs and PEDOT:PSS in energy conversion and storage devices. J. Mater. Sci. Mater. Electron..

[11] Tsai T.C., Chang H.C., Chen C.H., Whang W.T. (2011). Widely variable Seebeck coefficient and enhanced thermoelectric power of PEDOT:PSS films by blending thermal decomposable ammonium formate. Org. Electron. Phys. Mater. Appl..

[12] Kim J., Jang J.G., Hong J.I., Kim S.H., Kwak J. (2016). Sulfuric acid vapor treatment for enhancing the thermoelectric properties of PEDOT:PSS thin-films. J. Mater. Sci. Mater. Electron..

[13] Saxena N., Keilhofer J., Maurya A.K., Fortunato G., Overbeck J. (2018). Facile Optimization of Thermoelectric Properties in PEDOT:PSS Thin Films through Acido-Base and Redox Dedoping Using Readily Available Salts. ACS Appl. Energy Mater..

[14] Park H., Lee S.H., Kim F.S., Choi H.H., Cheong I.W., Kim J.H. (2014). Enhanced thermoelectric properties of PEDOT:PSS nanofilms by a chemical dedoping process. J. Mater. Chem. A.

[15] Mengistie D.A., Chen C.H., Boopathi K.M., Pranoto F.W., Li L.J., Chu C.W. (2015). Enhanced thermoelectric performance of PEDOT:PSS flexible bulky papers by treatment with secondary dopants. ACS Appl. Mater. Interfaces.

[16] Kim G.-H., Shao L., Zhang K., Pipe K.P. (2013). Engineered doping of organic semiconductors for enhanced thermoelectric efficiency. Nat. Mater..

[17] Luo J., Billep D., Waechtler T., Otto T., Toader M., Gordan O., Sheremet E., Martin J., Hietschold M., Zahn D.R.T. (2013). Enhancement of the thermoelectric properties of PEDOT:PSS thin films by post-treatment. J. Mater. Chem. A.

[18] Döbbelin M., Marcilla R., Salsamendi M., Pozo-Gonzalo C., Carrasco P.M., Pomposo J.A., Mecerreyes D. (2007). Influence of Ionic Liquids on the Electrical Conductivity and Morphology of PEDOT:PSS Films. Chem. Mater..

[19] Kee S., Kim N., Kim B.S., Park S., Jang Y.H., Lee S.H., Kim J., Kim J., Kwon S., Lee K. (2016). Controlling Molecular Ordering in Aqueous Conducting Polymers Using Ionic Liquids. Adv. Mater..

[20] Zhu Z., Liu C., Jiang Q., Shi H., Jiang F., Xu J., Xiong J., Liu E. (2015). Optimizing the thermoelectric properties of PEDOT:PSS films by combining organic co-solvents with inorganic base. J. Mater. Sci. Mater. Electron..

[21] Massonnet N., Carella A., Jaudouin O., Rannou P., Laval G., Celle C., Simonato J.-P. (2014). Improvement of the Seebeck coefficient of PEDOT:PSS by chemical reduction combined with a novel method for its transfer using free-standing thin films. J. Mater. Chem. C.

[22] Badre C., Marquant L., Alsayed A.M., Hough L.A. (2012). Highly Conductive Poly (3,4-ethylenedioxythiophene ):Poly(styrenesulfonate) Films Using 1-Ethyl-3-methylimidazolium Tetracyanoborate Ionic Liquid. Adv. Funct. Mater..

[23] Kim J., Kumar R., Bandodkar A.J., Wang J. (2017). Advanced Materials for Printed Wearable Electrochemical Devices: A Review. Adv. Electron. Mater..

[24] Chou T.R., Chen S.H., Chiang Y.T., Lin Y.T., Chao C.Y. (2015). Highly Conductive PEDOT:PSS Film by Post-Treatment with Dimethyl Sulfoxide for ITO-Free Liquid Crystal Display. Mol. Cryst. Liq. Cryst..

[25] Wang J., Cai K., Shen S. (2015). A facile chemical reduction approach for effectively tuning thermoelectric properties of PEDOT films. Org. Electron..

[26] Wang X., Meng F., Tang H., Gao Z., Li S., Jiang F., Xu J. (2017). An effective dual-solvent treatment for improving the thermoelectric property of PEDOT:PSS with white graphene. J. Mater. Sci..

[27] Beretta D., Barker A.J., Maqueira-Albo I., Calloni A., Bussetti G., Dell’Erba G., Luzio A., Duò L., Petrozza A., Lanzani G. (2017). Thermoelectric Properties of Highly Conductive Poly(3,4-ethylenedioxythiophene) Polystyrene Sulfonate Printed Thin Films. ACS Appl. Mater. Interfaces.

[28] Fan Z., Du D., Yao H., Ouyang J. (2017). Higher PEDOT Molecular Weight Giving Rise to Higher Thermoelectric Property of PEDOT:PSS: A Comparative Study of Clevios P and Clevios PH1000. ACS Appl. Mater. Interfaces.

[29] Zhang X., Wu J., Wang J., Zhang J., Yang Q. (2016). Solar Energy Materials & Solar Cells Highly conductive PEDOT: PSS transparent electrode prepared by a post-spin-rinsing method for ef fi cient ITO-free polymer solar cells. Sol. Energy Mater. Sol. Cells.

[30] Photovoltaics I.O. (2014). Unraveling the Enhanced Electrical Conductivity of PEDOT:PSS Thin Films for Unraveling the Enhanced Electrical Conductivity of PEDOT:PSS Thin Films for ITO-Free Organic Photovoltaics. IEEE Photonics J..

[31] Lenz A., Kariis H., Pohl A., Persson P., Ojamäe L. (2011). The electronic structure and reflectivity of PEDOT: PSS from density functional theory. Chem. Phys..

[32] Hwang J.S., Oh T.H., Kim S.H., Han S.S., Lee S.J., Lee S.G., Lee Y.J., Jang S.S. (2015). Effect of solvent on electrical conductivity and gas sensitivity of PEDOT: PSS polymer composite films. J. Appl. Polym. Sci..

[33] Wang X., Li M., Feng G., Ge M. (2020). On the mechanism of conductivity enhancement in PEDOT:PSS/PVA blend fiber induced by UV-light irradiation. Appl. Phys. A.

[34] Saghaei J., Fallahzadeh A., Yousefi M.H. (2015). Highly Conductive Poly(3,4-ethylenedioxythiophene):Poly(styrenesulfonate) Films Using 1-Ethyl-3-methylimidazolium Tetracyanoborate Ionic Liquid. Org. Electron. Phys. Mater. Appl..

[35] Teo M.Y., Kim N., Kee S., Kim B.S., Kim G., Hong S., Jung S., Lee K. (2017). Highly stretchable and highly conductive PEDOT:PSS/Ionic liquid composite transparent electrodes for solution-processed stretchable electronics. ACS Appl. Mater. Interfaces.

[36] Li Q., Yang J., Chen S., Zou J., Xie W., Zeng X. (2017). Highly Conductive PEDOT:PSS Transparent Hole Transporting Layer with Solvent Treatment for High Performance Silicon/Organic Hybrid Solar Cells. Nanoscale Res. Lett..

[37] Oh H.J., Jang J.G., Kim J.-G., Hong J.-I., Kim J., Kwak J., Kim S.H., Shin S. (2017). Structural and Morphological Evolution for Water-resistant Organic Thermoelectrics. Sci. Rep..

[38] Greczynski G., Kugler T., Salaneck W.R. (1999). Characterization of the PEDOT-PSS system by means of X-ray and ultraviolet photoelectron spectroscopy. Thin Solid Films.

[39] Mengistie D.A., Ibrahem M.A., Wang P., Chu C. (2014). Highly conductive PEDOT:PSS treated with formic acid for ITO-free polymer solar cells Highly Conductive PEDOT:PSS Treated with Formic Acid for ITO-Free Polymer Solar Cells. ACS Appl. Mater. Interfaces.

[40] Khan M.A., Armes S.P., Perruchot C., Ouamara H., Chehimi M.M., Greaves S.J., Watts J.F. (2000). Surface Characterization of Poly(3,4-ethylenedioxythiophene)-Coated Latexes by X-ray Photoelectron Spectroscopy. Langmuir.

[41] Keppler A., Himmerlich M., Ikari T., Marschewski M., Pachomow E., Höfft O., Maus-Friedrichs W., Endres F., Krischok S. (2011). Changes of the near-surface chemical composition of the 1-ethyl-3-methylimidazolium bis(trifluoromethylsulfonyl)imide room temperature ionic liquid under the influence of irradiation. Phys. Chem. Chem. Phys..

[42] Wei Q., Mukaida M., Naitoh Y., Ishida T. (2013). Morphological Change and Mobility Enhancement in PEDOT:PSS by Adding Co-solvents. Adv. Mater..

[43] De Kok M.M., Buechel M., Vulto S.I.E., van de Weijer P., Meulenkamp E.A., de Winter S.H.P.M., Mank A.J.G., Vorstenbosch H.J.M., Weijtens C.H.L., van Elsbergen V. (2004). Modification of PEDOT:PSS as hole injection layer in polymer LEDs. Phys. Status Solidi.

