# Supplementary material for: Enhanced Electrical Conductivity and Seebeck Coefficient in PEDOT:PSS via a Two-Step Ionic liquid and NaBH4 Treatment for Organic Thermoelectrics
Source: Polymers (Basel). 2020 Mar 3;12(3):559. doi: 10.3390/polym12030559 (PMC7182861; doi:10.3390/polym12030559)
Supplement: Supplementary file 1 [file polymers-12-00559-s001.pdf]

polymers

MDPI

Supplementary Information

Enhanced electrical conductivity and Seebeck coefficient in PEDOT:PSS via a two-step Ionic liquid & $NaBH_4$ treatment.

Jonathan Atoyo[1], Matthew R. Burton[2], James McGettrick[3] and Matthew J Carnie[1]†

Materials Research Centre, College of Engineering, SPECIFIC Swansea University, Bay Campus, Swansea SA1 8EN, United Kingdom

Introduction

This document includes all relevant materials in support of the paper

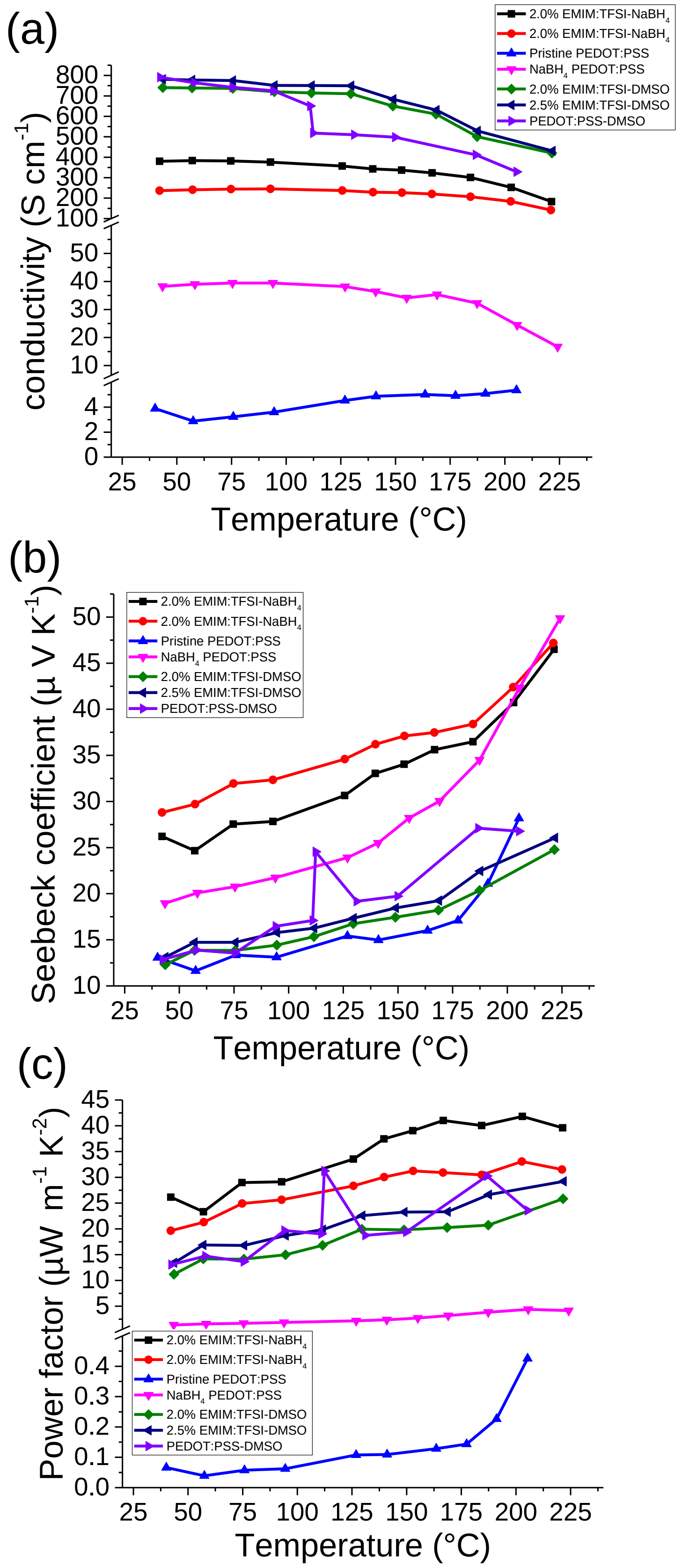

Fig. S1, depicting thermoelectric data of EMIM:TFSI, EMIM:TFSI-NABH$_4$ and DMSO controls

As depicted in Fig. S1 and Fig. 2, it's clear that the presence of DMSO is not responsible for the improved Seebeck coefficient in the films but instead NaBH$_4$.

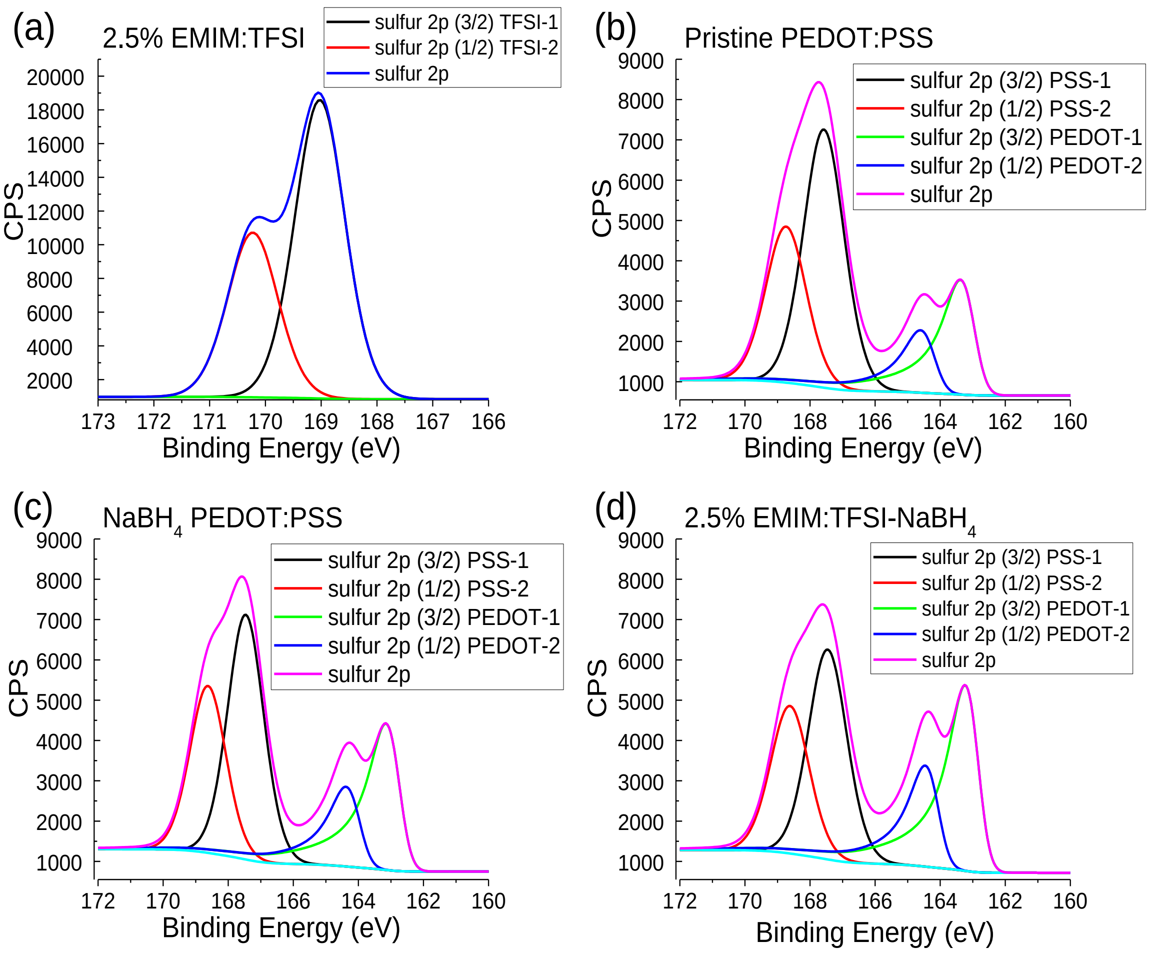

Fig. S2 represents the deconvoluted XPS spectra of 4 films, (a) denotes 2.5 % EMIM:TFSI film, (b) denotes Pristine PEDOT:PSS, (C) corresponds to the $NaBH_4$ Pristine PEDOT:PSS film, meanwhile (d) corresponds to the 2.5 % EMIM:TFSI NaBH4 film

Table S1. Sulphur 2p peak position and quantification data

**Sample**	**Peak name**	**Binding Energy (eV)**	**Concentration (%)**	**Total % Concentration**
2.5% EMIM:TFSI	Sulphur $2p_{(3/2)}$ TFSI-1	169.02	64.44	
	Sulphur $2p_{(1/2)}$ TFSI-2	170.23	35.56	100
Pristine PEDOT:PSS	Sulphur $2p_{(3/2)}$ PSS-1	167.58	42.57	
	Sulphur $2p_{(1/2)}$ PSS-2	168.74	25.74	68.31
	Sulphur $2p_{(3/2)}$ PEDOT-1	163.17	20.52	
	Sulphur $2p_{(1/2)}$ PEDOT-2	164.39	11.17	31.69
$NaBH_4$ PEDOT:PSS	Sulphur $2p_{(3/2)}$ PSS-1	167.46	36	
	Sulphur $2p_{(1/2)}$ PSS-2	168.62	24.55	60.55
	Sulphur $2p_{(3/2)}$ PEDOT-1	162.95	25.54	
	Sulphur $2p_{(1/2)}$ PEDOT-2	164.18	13.91	38.73
2.5% EMIM:TFSI-$NaBH_4$	Sulphur $2p_{(3/2)}$ PSS-1	167.46	31.45	
	Sulphur $2p_{(1/2)}$ PSS-2	168.62	22.12	53.57
	Sulphur $2p_{(3/2)}$ PEDOT-1	163.03	30.07	

	Sulphur 2p(1/2) PEDOT-2	164.26	16.37	46.44

Table S1, and Fig. S2 depict the deconvoluted peaks for 4 different films (also shown in Fig. 5(b) used to analyse the effect of EMIM:TFSI on the PSS to PEDOT ratio. For the pristine PEDOT:PSS film the PSS spin-orbital sulphur coupling (S $2p_{3/2}$) and (S$2p_{1/2}$) is depicted at 167.58 (eV) and 168.74 (eV) respectively.[1,2] As expressed in Fig 5(a) is the 2.5% EMIM:TFSI film, meanwhile 5(b) denotes Pristine PEDOT:PSS, (c) corresponds to the $NaBH_4$ Pristine PEDOT:PSS film, and (d) corresponds to the 2.5% EMIM:TFSI-$NaBH_4$ film. The (S $2p_{3/2}$) and (S $2p_{1/2}$) is seen at 163.17 eV and 164.39 eV for the sulphur corresponding to PEDOT in the Pristine PEDOT:PSS film.[3,4] It is clear that Pristine PEDOT:PSS has the highest PSS to PEDOT ratio. This is contrasted by the 2.5% EMIM:TFSI-$NaBH_4$ film with the lowest PSS to PEDOT ratio showing that EMIM:TFSI presence has a significant selective removal of PSS which can explain the improved electrical conductivity.

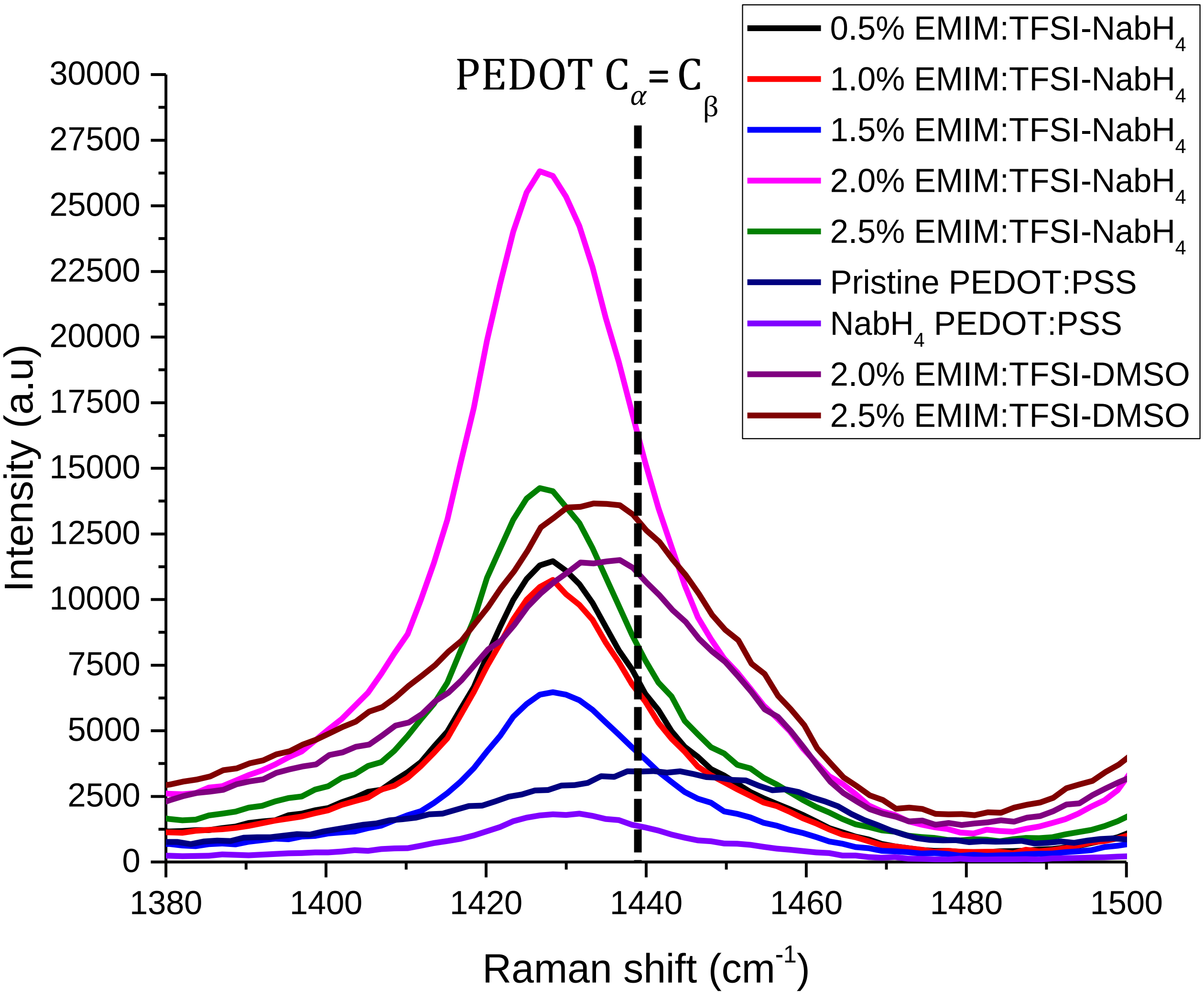

Fig. S3. Raman spectra depicting ionic liquid films treated with DMSO, post treatment without $NaBH_4$ to show that NaBH4 presence is the major cause for the increased red shift

As depicted in Fig. S3, the DMSO controls show a red shift which indicates a benzoid to quinoid conformational change for the thiophene molecules. However, relative to the $NaBH_4$ samples it is not as large indicating that the presence of DMSO in the $NaBH_4$ treated films is not the cause but rather the presence of $NaBH_4$ may be the cause of the large shift.

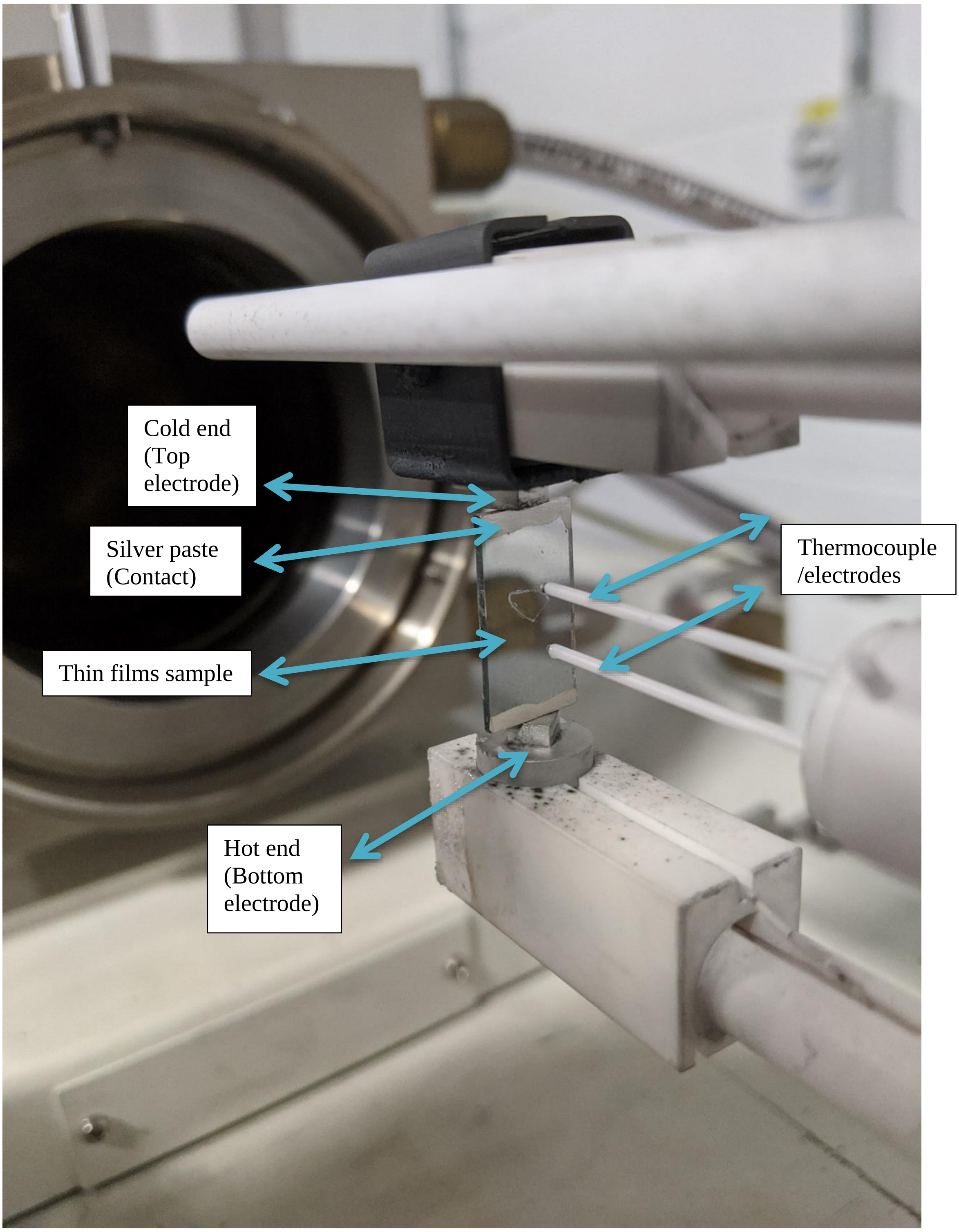

Fig. S4. ZEM-3 set up depicting electrodes and film measurement for Seebeck coefficient and electrical conductivity

References

1. Online, V.A., Mengistie, D.A., Wang, P., and Chu, C. (2013). E ff ect of molecular weight of additives on the conductivity of PEDOT : PSS and e ffi ciency for ITO-free. 9907–9915.

2. Vempati, S., Ertas, Y., Celebioglu, A., and Uyar, T. (2017). Tuning the degree of oxidation and electron delocalization of poly(3,4-ethylenedioxythiophene):poly(styrenesulfonate) with solid-electrolyte.

Appl. Surf. Sci. *419*, 770–777.

3. Yan, H., and Okuzaki, H. (2009). Effect of solvent on PEDOT / PSS nanometer-scaled thin films : XPS and STEM / AFM studies. *159*, 2225–2228.

4. Greczynski, G., Kugler, T., and Salaneck, W.R. (1999). Characterization of the PEDOT-PSS system by means of X-ray and ultraviolet photoelectron spectroscopy. *354*, 129–135.

© 2020 by the authors. Submitted for possible open access publication under the terms and conditions of the Creative Commons Attribution (CC BY) license (http://creativecommons.org/licenses/by/4.0/).
